# A multifactorial interdisciplinary intervention to prevent functional and mobility decline for more participation in (pre-)frail community-dwelling older adults (PromeTheus): study protocol for a multicenter randomized controlled trial

**DOI:** 10.1186/s12877-022-02783-4

**Published:** 2022-02-14

**Authors:** Christian Werner, Nacera Wolf-Belala, Corinna Nerz, Bastian Abel, Tobias Braun, Christian Grüneberg, Christian Thiel, Gisela Büchele, Reiner Muche, Ingrid Hendlmeier, Martina Schäufele, Judith Dams, Hans-Helmut König, Jürgen M. Bauer, Michael Denkinger, Kilian Rapp

**Affiliations:** 1 Center for Geriatric Medicine, Agaplesion Bethanien Hospital Heidelberg, Heidelberg University, Heidelberg, Germany; 2 Institute for Geriatric Research, Ulm University, Ulm, Germany; 3 Department of Clinical Gerontology, Robert-Bosch-Hospital, Stuttgart, Germany; 4 Division of Physiotherapy, Department of Applied Health Sciences, Hochschule für Gesundheit Bochum (University of Applied Sciences), Bochum, Germany; 5 Institute of Epidemiology and Medical Biometry, Ulm University, Ulm, Germany; 6 Department of Social Work, University of Applied Sciences, Mannheim, Germany; 7 Department of Health Economics and Health Services Research, University Medical Center Hamburg-Eppendorf, Hamburg, Germany; 8 Agaplesion Bethesda Clinic, Ulm, Germany

**Keywords:** Functional performance, Mobility, Exercise, Prevention, Frailty, Participation, Randomized controlled trial, Economic evaluation, Process evaluation, Community healthcare, Multifactorial, Implementation

## Abstract

**Background:**

Age-related decline in physical capacity can lead to frailty, associated with an increased vulnerability to adverse health outcomes and greater healthcare utilization. In an aging population, effective strategies to prevent physical decline and frailty, and preserve independence are needed. Prevention programs for vulnerable community-dwelling older adults are, however, often not yet established and implemented in routine practice. Research on the feasibility, implementation, and (cost-)effectiveness of multifactorial, interdisciplinary intervention programs that take advantage of available services of healthcare providers is also limited. The main aim of this study is to evaluate the effectiveness of such an intervention program (PromeTheus) to prevent functional and mobility decline for more participation in community-dwelling (pre-)frail older adults.

**Methods:**

The study is designed as a three-center, randomized controlled trial with a 12-month intervention period. Four hundred community-dwelling (pre-)frail (Clinical Frailty Scale score 4–6) older adults (≥70 years) will be randomized in a 1:1 ratio to the intervention group (IG) or the control group (CG). The IG will receive the PromeTheus program consisting of obligatory home-based physical exercises (Weight-bearing Exercise for Better Balance) accompanied by physiotherapists and facultative counseling services (person-environment-fit, coping with everyday life, nutrition, group-based activities) delivered via existing healthcare structures (e.g., social workers, nutritionists). The CG will receive usual care and a one-time counseling session on recommendations for physical activity and nutrition. Primary outcomes assessed at months 6 and 12 are the function component of the Late-Life Function and Disability Instrument and the University of Alabama at Birmingham Life-Space Assessment. Secondary outcomes are disability, physical capacity and activity, frailty, nutritional status, falls, fear of falling, health status, and psychosocial components. Process and economic evaluations are also conducted. Primary statistical analyses will be based on the intention-to-treat principle.

**Discussion:**

Compared to usual care, the PromeTheus program is expected to result in higher function and mobility, greater independence and lower need for care, and more participation. As the PromeTheus program draws on existing German healthcare structures, its large-scale translation and delivery will be feasible, if evidence of (cost-)effectiveness and successful implementation can be demonstrated.

**Trial registration:**

German Clinical Trials Register, . Registered on March 11, 2021.

## Background

The frailty syndrome is characterized by decreased physiological reserves and resistance to stressors that result in increased vulnerability to adverse health outcomes [1]. Frailty can also be described as the transitional stage in the cascade of functional decline from independence to disability [2]. (Pre-)frail older persons are at increased risk for limitations in physical functioning and mobility, falls, loneliness, lower social participation and quality of life, disability, hospitalization, institutionalization, and mortality when compared with robust persons [1, 3–7]. Onset and severity of frailty have also high economic relevance, as both are associated with greater healthcare and social service costs [8, 9]. (Pre-)frailty is common among community-dwelling adults aged 70 years and older, with prevalence rates of 20% for frailty and 49% for pre-frailty [10]. Due to the demographic change, these prevalence rates are expected to further increase [11], which is also likely to result in an increase in disability and need for health and social care resources. Frailty has become one of the most serious public health issues [12, 13]. In an aging population, effective and early initiated intervention strategies to prevent or reduce functional decline, the onset and progression of frailty, disability, and the need for care are paramount for both the older individual and the healthcare system [14, 15].

Modifiable risk factors for frailty and functional disability should form the basis for public health and prevention strategies [16]. These factors cover a wide range of physical, lifestyle-related, social and psychological aspects [17–21]. In this regard, physical inactivity has been recognized as one of the major risk factors for functional decline and frailty onset or progression [12, 21, 22]. Meanwhile, multifactorial, interdisciplinary interventions with physical exercise as the main component, supplemented by additional nutritional, environmental, and psychosocial components, have been shown to be effective in preventing and reducing functional decline and frailty in community-dwelling older adults [23, 24].

Current primary healthcare services in Germany and many other high-income countries are generally fragmented, reactive, disease-oriented and underfunded, especially in the community setting [25, 26]. General practitioners (GPs) are positioned to screen community-dwelling older adults early in the trajectory of functional decline, identify those who are (pre-)frail, and initiate strategies to prevent or counteract the potential progression to frailty and disability. However, GPs have only limited access to community-based primary or secondary prevention programs for vulnerable older adults, as evidence-based programs are often lacking in daily practice. It has become evident that few comprehensive multifactorial, interdisciplinary intervention programs have been integrated to date to prevent functional decline and frailty in the community and at the interface between primary and secondary care of older adults [23, 27]. Evidence for the (cost-)effectiveness of such interventions is also limited [12, 22, 28, 29].

In Germany, physiotherapy is currently the most community-based service prescribed by GPs to prevent or reduce functional decline and frailty. However, such therapy, which is usually only episodic, seems to be too one-dimensional, insufficiently tailored to the complex care needs of (pre-)frail older people, and poorly focused on sustainability. Comprehensive community-based intervention programs for primary and secondary prevention in old age are currently not available and implemented in Germany. Inpatient geriatric rehabilitation as a multidisciplinary treatment to optimize function, promote activity, and preserve independence and social participation [30] is available but is mainly prescribed after hospitalization resulting from an acutely triggered impairment. Although there might be a number of benefits associated with such inpatient services, this setting is not only undesirable from a health economic perspective due to its high costs, but also from an individual point of view. Many older adults prefer to stay at home to “age in place” rather than be admitted to inpatient services [31]. Inpatient settings can also be associated with complications such as nosocomial infections [32] or an increased tendency to falls in unfamiliar surroundings [33], further increasing the need for community-based alternatives.

To prevent and manage frailty in community-dwelling older adults, it has been recommended to provide GPs with more support for implementing multifactorial, interdisciplinary interventions [16]. In this context, comprehensive intervention strategies should take advantage of already existing resources and structures of interdisciplinary healthcare providers, and community barriers to care services for frail older adults need to be removed. Research on the feasibility, implementation, and (cost-)effectiveness of such intervention strategies is, however, still limited [16, 23, 34].

The primary aim of this study is to compare the effectiveness of a 12-month multifactorial, interdisciplinary intervention program (PromeTheus) with usual care in preventing functional and mobility decline for more participation in community-dwelling (pre-)frail older adults. The PromeTheus program is based on the Frailty Intervention Trial (FIT) program from Australia [35], which has been shown to be (cost-)effective in reducing frailty, mobility disability, and fall risk factors [25, 36, 37] and will now be adapted to the German healthcare system. The major components of the PromeTheus program consist of an obligatory home-based physical exercise program accompanied by physiotherapists, and facultative counseling services (person-environment-fit, coping with everyday life, nutrition, group activities) delivered via already existing structures and stakeholders of German healthcare providers. Secondary aims are (1) to evaluate the effects of the PromeTheus program on physical capacity and activity, frailty, falls, nutritional status, health status and quality of life, health-related resource use, and psychosocial status, (2) to compare the cost-effectiveness of the PromeTheus program with that of usual care, and (3) to conduct a process evaluation according to the Medical Research Council (MRC) framework for evaluating complex interventions [15]. The main hypotheses of this study are that the PromeTheus program will result in higher function and mobility, greater independence and lower need for care, and more participation after the 12-month intervention period compared to usual care.

## Methods

### Study design

The study is designed as a three-center, assessor-blinded, randomized (1:1), controlled, parallel-group trial with a 12-month intervention period. Four hundred participants will be recruited from the communities at three study sites in the federal state of Baden-Wuerttemberg, Germany: (1) Robert-Bosch-Hospital (Stuttgart, Germany: *n* = 150), (2) Agaplesion Bethanien Hospital (Heidelberg, Germany: *n* = 150), and (3) Agaplesion Bethesda Clinic (Ulm, Germany: *n* = 100). This study protocol was reported in accordance with the SPIRIT (Standard Protocol Items: Recommendations for Interventional Trials) guidelines [38], and follows the CONSORT (Consolidated Standards for Reporting Trials) guidelines for transparent reporting of parallel group randomized trials [39].

### Eligibility criteria

Eligible participants are (pre-)frail (Clinical Frailty Scale [CFS] score = 4–6 points) older adults aged ≥70 years living at home or in assisted living facilities, insured with the largest health insurance company in the German federal state of Baden-Wuerttemberg (‘Allgemeine Ortskrankenkasse [AOK] Baden-Württemberg’), and able to walk ≥10 m with or without walking aid. An overview of all inclusion and exclusion criteria is provided in Table 1.

**Table 1:** Inclusion and exclusion criteria for study participation

Inclusion criteria	Exclusion criteria
• age ≥ 70 years • Clinical Frailty Scale score 4 (“very mild frailty”), 5 (“mildly frail”), or 6 (“moderately frail”) • living at home or assisted living • able to walk ≥10 m with or without walking aid	• able to walk ≥800 m without walking aid or breaks • cognitive impairment (Short Orientation-Memory-Concentration Test score > 10) • insufficient German language skills • visual acuity not sufficient to recognize study material • medical conditions: - heart failure (NYHA III-IV) - stroke within the last 6 months - Morbus Parkinson (Hoehn & Yahr Stage ≥3) - cancer, if currently under treatment (e.g., chemotherapy, radiation) or in an advanced stage - severe lung disease requiring (intermittent) oxygen supply - multiple sclerosis

### Intervention

**Fig. 1:**
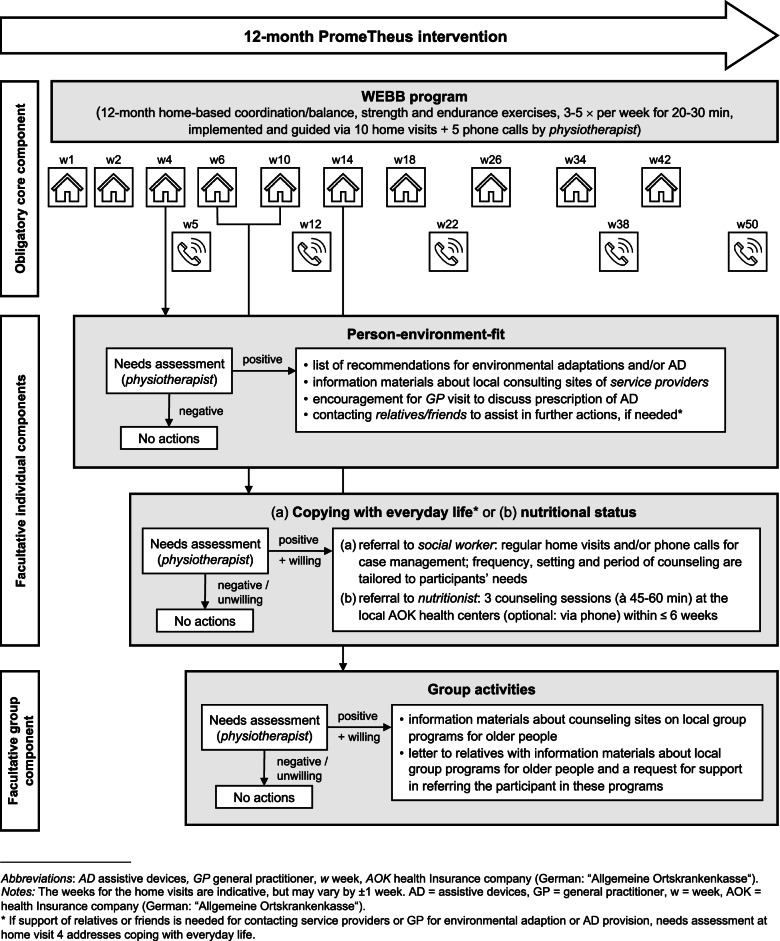
Flow of the PromeTheus intervention program

Participants in the IG will receive the multifactorial, interdisciplinary PromeTheus program for 12 months, which is based on the FIT program [35] and aims to reduce functional and mobility decline, maintain independence and improve participation. The physical exercise component is adopted identically as an obligatory core component from the FIT program. Additional intervention components of the FIT program on nutrition, psychological well-being, social engagement, and healthcare provision have been adapted to the German healthcare system and, if indicated, will be offered and implemented as facultative components through existing structures and stakeholders of healthcare providers. The PromeTheus program includes three components: (1) “Weight-bearing Exercise for Better Balance” (WEBB) program (→ obligatory core component) [40]; (2) counseling on person-environment-fit (i.e. environmental adaption, provision of assistive devices), nutritional intake, and/or coping with everyday life (→ facultative individual components), and (3) integration into long-term training and/or social-communicative group programs (→ facultative group component). It is coordinated by physiotherapists and primarily delivered at the participants’ homes. All intervention components are individually tailored to each participant based on standardized assessments conducted by the trained physiotherapists during the intervention program. Physiotherapists also receive a detailed trainer’s manual that contains all program information. Table 2 provides a systematic description of the intervention components of the PromeTheus program using the Template for Intervention Description and Replication (TIDieR) checklist [41], and Fig. 1 depicts the flow of the intervention.

**Table 2:** Description of the PromeTheus intervention using the TIDieR checklist [41]

Item	Description
1. Brief name	Prevention for more participation in old age (PromeTheus)
2. Why	Multifactorial, interdisciplinary interventions with physical exercise as the main component, supplemented by additional nutritional, environmental, functional, and psychosocial components, have been shown to be effective in frailty management. Evidence on the (cost-)effectiveness and the successful implementation of such interventions in routine practice via available healthcare structures is limited.
3. What: Materials	WEBB: German WEBB program manual (general training principles; pictorial and written exercise descriptions; exercise frequency, volume, duration, intensity, *and progression; Borg Rating of Perceived Exertion scale,* precautions and safety issues), workbook (training sheets with individual exercise prescriptions and training goals, training diaries, DEMMI progress chart, EARS, contact information of physiotherapists), training materials (weight vest, balance pad, anti-slip mat, objects to step over [e.g., book] and to grasp [e.g., water bottle], adhesive tape to make marks on the floor, cushion, table, chair) Counseling on person-environment-fit: 25-item checklist, information materials on local consulting sites of service providers (e.g., healthcare supply stores), pre-formulated cover letter to GP Counseling on coping with everyday life: 10-item screening tool, referral document to the social worker Nutritional counseling: MNA-SF, SNAQ, referral document to the nutritionist, 7-day nutritional protocol, brochure on malnutrition in old age Group component: information materials about local counseling sites on group offers for older people, information letter to the relatives with this information and a request for support in finding and referring participants to appropriate group programs The trainer manual with all information is also included in the participants’ WEBB program manual and workbook, with additional content and task descriptions of all 10 home visits and 5 phone calls, instructions for goal setting, motivational interviewing, and feedback provision on training progress.
4. What: Procedures	10 home visits and 5 phone calls by one physiotherapist Home visit 1: The PromeTheus program is introduced, all documents (WEBB program manual, workbook) and training materials are handed over and reviewed with the participants, participants’ physical capacity is assessed (DEMMI), an adequate training location in the home is identified, and a training plan with first exercises is worked out. Home visit 2: The exercises that were not yet introduced in home visit 1 are added to the training plan, existing exercises are adapted to increase the training stimulus, if necessary, and 2–4 training goals are defined. Subsequent home visits and phone calls: Training plans, exercise prescriptions and attainment of training goals are evaluated and adapted to the participants’ training progress. DEMMI is assessed at home visits 6 and 10. Home visits 3–5: Needs assessments for facultative individual intervention components (person-environment-fit, coping with everyday life, nutrition) are performed. 2nd intervention quarter: Needs assessments for the facultative group component. If there is a need and willingness for counseling, information materials on local consulting sites of service providers are provided (counseling on person-environment-fit), relatives are involved for support (referral to group activities), GP visit is encouraged (prescription of assistive devices), or participants are referred to the social workers (counseling on coping with everyday life) or nutritionists (nutritional counseling).
5. Who provided	Physiotherapists (WEBB), service providers at the local sites (counseling on person-environment-fit), AOK social workers with qualification as a care consultant (counseling on coping with everyday life), AOK nutritionists (nutritional counseling)
6. How	Intervention is provided in one-on-one situations in participants’ homes (WEBB, counseling on coping with everyday life), at the AOK facilities (nutritional counseling), or via phone (WEBB, counseling on coping with everyday life, nutritional counseling)
7. Where	Three study sites: Stuttgart, Heidelberg, and Ulm (Baden-Wuerttemberg, Germany)
	Primarily delivered in participants’ homes (WEBB, counseling on person-environment-fit and coping with everyday life), additional out-of-home appointments offered if needed and willing to (nutritional counseling at the AOK facilities in the city centers, group activities in the participants’ local area)
8. When and how much	WEBB: 10 home visits (week 1, 2, 4, 6, 10, 14, 18, 26, 34, 42) à 30–60 min, 5 phone calls (week 5, 7, 22, 38, 50) à 20 min, overall exercise prescription: 3–5 ×/week à 20–30 min Counseling on person-environment-fit (after week 3) and coping with everyday life (after week 4 or 5), group activities (start: 2nd intervention quartal), if needed and willing to: frequency, schedule, duration, etc. individually tailored to the participants’ needs/interests Nutritional counseling (after week 4 or 5), if needed and willing to: 3 sessions à 45–60 min within ≤6 weeks
9. Tailoring	WEBB: Individual tailoring of the exercise prescription (e.g., training frequency, intensity, volume) and training goals is constantly given at each home visit and a phone call by the physiotherapist. Facultative components (counseling on person-environment-fit, coping with everyday life, nutrition, and group activities) are provided based on individual participant needs.
10. Modifications	N/A
11. How well:Planned	Training adherence is assessed using self-reported training diaries and the EARS filled out by the participants after home visits 3, 6 and 8. Training diaries contain sheets with (1) check boxes for each day that participants mark differently for different types of exercises completed, and (2) blank spaces for each exercise in which participants document the execution, sets, repetitions, weight, and/or duration for each exercise Motivational and volitional techniques of behavior change are used to increase training adherence: provision of training information (WEBB program manual), regular home visits/phone calls for setting, reviewing and adapting individual training goals, barrier identification and problem-solving, self-monitoring by training diaries, motivational interviewing, provision of feedback on training progress.
12. How well: Actual	N/A

*Abbreviations*: *AOK* health insurance company (German: ‘Allgemeine Ortskrankenkasse’), *DEMMI* de Morton Mobility Index, *EARS* Exercise Adherence Rating Scale, *GP* general practitioner, *MNA-SF* Mini Nutritional Assessment – Short Form, *SNAQ* Simplified Nutritional Appetite Questionnaire, *WEBB* Weight-bearing Exercise for Better Balance

#### Obligatory core component: “Weight-bearing Exercise for Better Balance” (WEBB)

Participants of the IG will perform home-based exercises following the WEBB program, which has been designed to improve mobility, increase physical activity and prevent falls [40]. The WEBB program will be taught, accompanied, and regularly reviewed by physiotherapists in one-on-one sessions via 10 home visits and 5 phone calls over the 12-month intervention period. Participants will learn in what way, how long and often, and at what intensity to perform balance/coordination, strength, and endurance exercises on their own at home. All exercises are described in a German WEBB program manual, which is provided to the participants at the first home visit. Participants will be initially instructed on 2–3 days per week to perform (a) 3 × 10 repetitions of ≥2 balance/coordination exercises (e.g., tandem stand, move an object to a higher position, climb over obstacles), (b) 2 × 10–15 repetitions of ≥3 lower extremity strength exercises (e.g., chair lifting, calf raising, stair climbing) with a perceived exertion of 15 (hard) on the Borg Rating of Perceived Exertion scale [42], and (c) a 20–30-min endurance session (e.g., walking, bicycle ergometer, swimming; split into 10-min bouts if needed) at moderate intensity. These exercise prescriptions are further tailored to the participants’ individual physical capacities and training goals and are being documented on training sheets in a participant workbook at each home visit. Motivational and volitional techniques of behavior change (e.g., provision of a program manual; setting, reviewing and adapting individual training goals; barrier identification and problem-solving; self-monitoring by training diary, motivational interviewing, feedback provision on training progress) will be used to encourage participants to this training regularity [43, 44].

Two to four individual training goals will be elaborated, set, and documented together with the physiotherapists during the second home visit using the SMART (Specific, Measurable, Applicable, Realistic, Time-bound) criteria [45]. Goal attainment will be regularly evaluated during each home visit and phone call, with goals being adapted or new ones formulated as training progresses.

To adapt the training to the participants’ individual physical capacity and training progress, physiotherapists will regularly assess the de Morton Mobility Index (DEMMI [46, 47]). The DEMMI will also be used to provide and illustrate feedback to participants on their training progress by documenting the DEMMI scores collected in a progress chart in the participants’ workbook.

To promote and assess training adherence, participants are encouraged to self-monitor all training sessions in a training diary. Self-reported training adherence will also be measured using the Exercise Adherence Rating Scale (EARS [48]) after home visits 3, 6, and 8.

#### Facultative individual components

The need for individual counseling services is assessed by the physiotherapists within the first five home visits. In a first step, the need for a person-environment fit is assessed at home visit 3. In a second step, the need for counseling on nutrition and coping with everyday life is assessed at home visits 4 and 5, respectively (order depends on the relevance for the participant as estimated by the physiotherapist).

##### Person-environment-fit

The need for adaptions of the home environment and/or for the assistive device(s) will be assessed using a 25-item checklist on participants’ general situation (e.g., walking aids, hearing and vision), access to and mobility within the home, bathroom, toilet and kitchen equipment, and other aspects (e.g., bed height, lighting). If relevant needs for environmental adaptions and/or assistive device(s) are identified, participants receive a list of recommendations and information materials on local consulting sites of service providers (e.g., healthcare supply stores, municipal/non-profit counseling centers). If an assistive device is recommended, participants are also provided with a pre-formulated cover letter to their GP and are encouraged to use it as a basis to explore a potential prescription for the device with the GP.

##### Coping with everyday life

A 10-item screening tool will be used to assess the needs for self-management and psychosocial support in everyday life. The items address housework, correspondence, personal affairs, and out-of-home activities, as well as depressive symptoms and loneliness, social support, life satisfaction, caregiving and caregiver burden, healthcare proxy, and advance directives. If a need for support is identified for any of these items and the participant is willing to receive counseling for it, the physiotherapist refers the participant to a local social worker and forwards the results of the needs assessment. The social worker provides counseling on coping with everyday life as part of a case-management in social and health care, which is implemented through regular home visits and/or telephone calls. All counselors are state-approved social workers of the social service of the AOK health insurance, who have an additional qualification as long-term care counselors. The frequency and setting (home visit or phone call) of the counseling sessions and the total counseling period are not predefined but will be individually tailored to the specific needs and wishes of each participant.

##### Nutritional status

Needs assessments for nutritional counseling will be performed using the Mini Nutritional Assessment – Short Form (MNA-SF [49]) and the Simplified Nutritional Appetite Questionnaire (SNAQ [50]). Participants who meet the criteria for (risk of) malnutrition, defined by a MNA-SF score ≤ 11 points [49], and/or anorexia, defined by a SNAQ score ≤ 14 points [50], will be recommended and encouraged to take advantage of the nutritional counseling offered by nutritionists of the AOK health insurance. If participants are willing, physiotherapists refer them to the local nutritionist at the respective study site. In preparation for the nutritional counseling, participants complete a 7-day nutritional protocol and send it to the nutritionist, who also receives the results of the needs assessment (MNA-SF, SNAQ). The nutritional counseling will include a total of three sessions of 45 to 60 min (session 1: on-site at the AOK facilities, session 2 & 3: optionally also via phone), within 6 weeks after the need and willingness for nutritional counseling have been determined. It will focus on providing information on prevention and treatment strategies for malnutrition (e.g., adequate energy, protein, (micro-)nutrients and fluid intake, additional snacks, meal fortification). All participants who receive nutritional counseling will also be given a booklet targeting this information.

#### Facultative group component: group activities

Physiotherapists will conduct a semi-structured interview with participants beginning in the second quarter of the intervention to explore the need for referral to group programs (e.g., social-communicative, musical, creative-artistic, or physical activities) to address potential loneliness, promote participation, and maintain training routines. In this interview, the relevance and potential benefits of group activities will be explained and participants will be asked about, for example, activities they already attend, their interest in taking up additional activities, reasons for previously discontinued activities, and their knowledge of group activities in the local area. If they wish to engage in other activities or do not yet attend any but are willing to do so, information materials about local counseling sites on group offers for older people will be provided. Participants will also be asked for their consent to contact their relatives by mail with the information materials and a request for support in finding and referring them to appropriate group activities.

### Control group

Participants allocated to the CG receive usual care consisting of health and elderly care services routinely available to older people in the German healthcare system. In addition, they receive a handout with global recommendations on physical activity for health according to the World Health Organization [51] and on nutrition in old age according to the German Nutrition Society [52]. These recommendations are handed over and communicated by a physiotherapist in a one-time, 45-min counseling session at participants’ homes.

### Outcomes

All primary and secondary outcomes, screening parameters, and descriptive variables are listed in Table 3. Data on sociodemographics, medical and medication information, neuropsychological status, function and participation, physical capacity and mobility, frailty, physical activity status, health status and economics, psychosocial data, nutritional status, training adherence, and perceived exertion while exercising are assessed at varying time points. To ensure the highest possible standardization of assessments, all assessors receive extensive training in all aspects of screening and assessments before recruitment starts. All primary and secondary outcomes are assessed at participants’ homes.

**Table 3:** Overview of outcome measures, screening instruments, and descriptive measures over the course of the study

		PS	TS	T_0_	INT	T_1_	T_2_
	*Sociodemographics*						
	Age; birthday; sex; living conditions	X	X				
	Living alone or not; marital status; school-leaving qualification; years of education; academic grades; retirement date		X				
	German-speaking		X				
	*Medical and medication information*						
	Height; weight; body mass index			X	X		
	No medical contraindications for the intervention program	X					
	Vision impairment: “Are you able to read a newspaper or book, with or without visual aid?”		X				
S	Fall history and fall-related injuries in the past 3 and 6 months ^a^			X		X	X
	Prevalence of neurologic, pulmonary, or cardiac diseases		X				
	Fall calendar over 12 months				X		
S	Comorbidities incl. Treatment; cardiac issues or stroke in past 6 months; use of sedatives or anticonvulsants; number of hospital admissions in past 6 months			X		X	X
S	Medication use (type, dosage, frequency)			X		X	X
	*Neuropsychological status*						
S	Exhaustion: Center for Epidemiologic Studies Depression Scale, 2-item version [53] ^c^			X		X	X
	Cognitive status: Short Orientation-Memory-Concentration Test [54]			X			
S	Fear of falling: Short Falls Efficacy Scale International [55]			X		X	X
	*Function, mobility, and participation*						
P	Late-Life Function and Disability Instrument – function component [56]			X		X	X
P	University of Alabama at Birmingham Life-Space Assessment [57, 58]			X		X	X
S	Short Form of the Late-Life Function and Disability Instrument – disability component [56]			X		X	X
	*Physical capacity*						
	Walking ability > 10 m (with/without walking aid)	X					
S	Short Physical Performance Battery [59] ^c^			X		X	X
S	Handgrip strength: dynamometer ^c^			X		X	X
	De Morton Mobility Index [46, 47]				X		
	*Frailty*						
S	Clinical Frailty Scale [60]	X		X		X	X
S	Fried frailty phenotype [1]			X		X	X
	*Physical activity*						
S	German Physical Activity Questionnaire 50+ [61] ^c^			X		X	X
S	Sensor-based physical activity (body postures, [in-]active states, transfers, walking activity)						X
	*Health status and economics*						
	Subjective health: “Compared with other people in your age group, how would you rate your personal health?”		X				
S	Health-related quality of life: EuroQol-5-Dimension 5-Level, EuroQol visual analog scale [62]			X		X	X
S	Health-related resource use: adapted version of the questionnaire for the use of medical and non-medical services in old age (FIMA) [63]			X		X	X
	*Psychosocial status*						
S	Self-Efficacy: Generalized Self-Efficacy Scale [64] ^b^			X		X	X
S	Social network: Lubben Social Network Scale [65]			X		X	X
S	Loneliness: UCLA 3-item loneliness scale [66]			X		X	X
S	Affect: Visual Analogue Scale [67]			X		X	X
S	Motivation: Behavioral Regulation in Exercise Questionnaire [68, 69] ^b^			X		X	X
	*Nutritional status*						
S	Mini Nutritional Assessment – Short Form [10] ^c^			X	X	X	X
	Simplified Nutritional Appetite Questionnaire [50]				X		
	*Training adherence and exertion*						
	Training diary (training days, sessions, sets, repetitions, and weights/duration) ^b^				X		
	Exercise Adherence Rating Scale [48] ^b^				X		
	Borg Rating of Perceived Exertion scale [42] ^b^				X		

^a^ part of quarterly-returned fall calendar over 12 months

^b^ included in process evaluation

^c^ as part of frailty assessment in accordance to the Fried frailty phenotype [1]

*Abbreviations*: *T*_*0*_ baseline assessment, *T*_*1*_ 6-month assessment, *T*_*2*_ 12-month assessment, *INT* within-intervention assessments, *PS* pre-screening by general practitioner, *P* primary outcome measure (or part of it), *S* secondary outcome measure, *TS* telephone screening

#### Primary outcomes

The primary outcome measure will be the function component of the Late-Life Function and Disability Instrument (LLFDI-FC) [70]. The LLFDI-FC assesses self-reported difficulties in performing 32 physical activities on three functional domains: (1) upper extremity (7 items), (2) basic lower extremity (14 items), and (3) advanced lower extremity functioning (14 items). All LLFDI-FC items are scored on a rating scale of 1 (“none difficulty”) to 5 points (1 = “cannot do”). Final scoring includes an overall LLFDI-FC function score and three separate domain scores, all ranging from 0 to 100 points, with higher scores indicating higher physical functioning. The LLFDI-FC is a well-established patient-reported outcome measure (PROM) to assess physical functioning in community-dwelling older adults, and has been demonstrated to have good-to-excellent construct/predictive validity and test-retest reliability and to be sensitive to change [56, 70, 71].

The University of Alabama at Birmingham Life-Space Assessment (LSA) [57, 58] will be used as the second primary outcome measure. The LSA is a self-report instrument to assess the extent of an individual’s real-life mobility within one’s environment, categorized into six hierarchically structured life-space zones (bedroom, home, outside the home, neighborhood, hometown, beyond the hometown), and the frequency (< 1×/week, 1–3×/week, 4–6×/week, daily) and independence of mobility within each zone (personal assistance, equipment only, no assistance) in the previous 4 weeks prior to the assessment. The LSA total score ranging from 0 points (“totally bed-bound”) to 120 points (“traveled out of town every day without any assistance”) is calculated based on the life-space zones, the frequency of attaining each zone, and the degree of independence in achieving each zone. Feasibility, construct validity, test-retest reliability, and sensitivity to change of the LSA have been established previously in community-dwelling older adults [57, 58, 72, 73].

#### Secondary outcomes

The disability component of the short form of the LLDFI will be used to collect information about the frequency of participation in certain activities and associated limitations while performing them [74, 75].

Physical capacity will be assessed using the Short Physical Performance Battery, which includes a standing balance, 4-m gait speed, and repeated chair stand test [59]. Handgrip strength will be measured with a JAMAR digital hand dynamometer.

Self-reported physical activity will be assessed via the German Physical Activity Questionnaire 50+, which addresses the amount of time spent on physical activities during the last month in the domains of housework, gardening, leisure time, sports, and work [61]. At the 12-month assessment, physical activity will also be objectively measured over 7 days using a small (23.0 × 32.5 × 8.9 mm) and lightweight (11 g), water-resistant 6-axis inertial measurement unit (AX6, Axivity Ltd., Newcastle, United Kingdom) fixed at the lower back using self-adhesive fixing foil. Outcomes on body postures (e.g., sitting, lying, standing), (in-)active states, transfers, and walking activity will be derived from raw data using the most recently available validated algorithms [76].

Falls [77] will be continuously recorded by the participants for each week over the 12-month study period using a fall calendar that contains information on date, time, and place of the falls, injuries, and activity *prior to falling* and will be mailed to the study centers quarterly. Fear of falling will be assessed by the short version of the Falls Efficacy Scale-International [55].

The nutritional status will be screened by the MNA-SF, which includes six items about food intake, weight loss, mobility, acute disease or psychological stress, neuropsychological problems, and body mass index [49].

Frailty will be assessed by the CFS [60] and the five criteria of the Fried frailty phenotype (unintentional weight loss, exhaustion, weakness, slowness, low physical activity) [1]. The 5-level version of the EuroQol-5-Dimension (EQ-5D-5L) questionnaire and the EuroQol visual analog scale will be used to measure self-reported health-related quality of life and health status [62].

Psychosocial components assessed in the study will include self-efficacy (Generalized Self-Efficacy Scale) [64]), social network (6-item Lubben Social Network Scale [65]), loneliness (UCLA 3-item loneliness scale [78]), affect (VAS [67]), and motivational quality (Behavioral Regulation in Exercise Questionnaire [68, 69]).

#### Health economic evaluation

In the health economic evaluation, both the intervention costs and the costs for the utilization of health services will be taken into account. Thereby, intervention costs will consist of personal costs and direct (non-)medical costs that are directly related to the intervention. The personnel costs due to time spent for the intervention will be derived from average German wages of the professional groups involved. The health service utilization will be assessed with an adapted version of the FIMA questionnaire (German: *‘Fragebogen zur Inanspruchnahme* medizinischer und nicht-medizinischer Versorgungsleistungen im Alter’) covering direct medical costs of formal healthcare services (e.g., in−/outpatient treatment, rehabilitation services, medications) and direct non-medical costs of informal care (e.g., support in activities of daily living from family members, friends or neighbors) [63]. Health service utilization will be monetary values based on standardized unit costs [79]. All costs will be collected for the previous 6 months at the baseline, 6- and 12-month assessments and will be compared between the IG and CG.

For the cost-effectiveness analysis health effects will be measured using quality-adjusted life years (QALYs [80]) based on the EQ-5D-5L [62]. For this analysis, the incremental cost-effectiveness ratio will be calculated as the ratio between the difference in costs and the difference in QALYs between the study groups. In addition, cost-effectiveness acceptability curves will be constructed based on net-benefit regressions to account for statistical uncertainty.

#### Process evaluation

A process evaluation will be conducted in accordance with the MRC framework for evaluating complex interventions [15] to elicit the conditions of success or failure of the PromeTheus program and its implementation, and to identify key influencing factors for differential intervention outcomes. It will examine (1) whether the PromeTheus program is implemented as intended (*fidelity*), (2) to what extent the intervention is given (e.g., training units of the intervention team, duration of home-visits) (*dose*), (3) whether the potential addressees are reached (*reach*), (4).

whether any adaptations during the implementation process are necessary *(adaptations),* (5) to what extent the participants adhere to the program, and how they experience it (*participants response*) and (6) which contextual factors hinder or facilitate the implementation and intervention outcomes (*context*). Data collection and analysis of the process evaluation will be based on a mixed-method approach. Quantitative data will be collected from documentation sheets of the home visits, training diaries and workbooks, questionnaires on participants’ motivation, self-efficacy and satisfaction, protocols of the counseling sessions, and process audits for the counseling services (see also Table 3). Qualitative data will be extracted from interviews and focus groups and interviews with physiotherapists, social workers, nutritionists, and IG participants.

### Participant timeline

The three-stage screening procedure includes (1) a pre-screening by GPs on-site at their private practice (5–10 min), (2) a subsequent telephone screening by study staff members (15–20 min), and (3) a cognitive screening at the potential participant’s home before baseline assessment (5 min). If eligibility for participation is confirmed, baseline assessment will be performed directly after the cognitive screening. At this baseline assessment, participants will receive quarterly fall calendars for a total duration of 1 year. After baseline assessment, participants will be randomized into the IG or CG. During the 12-month study period, the IG will take part in the PromeTheus program, while the CG will receive usual care and the one-time counseling session at home. Assessments will take place 6 months (±2 weeks) and 12 months (±2 weeks) after the intervention starts. All assessment sessions will last about 1.5 h each.

### Sample size

The sample size calculation was performed for a group comparison at 12 months (T_2_) in the LLFDI-FC score as the primary outcome of the study. Based on the baseline LLFDI-FC score of 69 points with a standard deviation of 15 points reported in the LiFE study [81] for an elderly population similar in frailty to the target sample of the PromeTheus study, and the minimal clinically important difference of 5 points reported for the LLFDI-FC in community-dwelling older adults with mobility limitations [82], 143 participants per group are required to achieve a power of 80% at a significance level of 5% using a 2-tailed *t*-test for independent samples. Considering a dropout rate of 20%, as observed in the LiFE study [81], the sample size increases to 179 participants for each group. To account for additional variance due to the multicenter study design, the sample size was further increased by 10%, resulting in a final sample size of 199 participants (rounded *n* = 200) per group.

### Recruitment

**Fig. 2:**
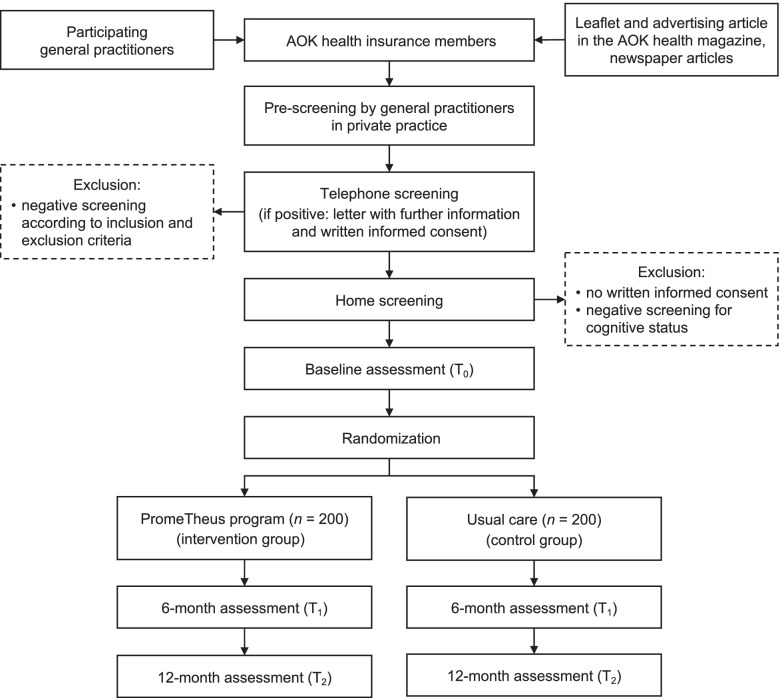
Flow chart of the recruitment, screening, allocation, and assessment processes

The recruitment, screening, allocation and assessment processes of participants throughout the study are shown in Fig. 2. Participant recruitment is primarily carried out by participating GPs. Local GPs at each study site are contacted by informative letter, explaining the background, contents, procedures and aims of the PromeTheus study, the role and responsibilities of the GPs in the study procedure, and the registration procedure for participation (January 2021). After written registration, the documents required for the recruitment and pre-screening process are mailed by the study centers to the participating GPs. The primary recruitment strategy is performed during a routine patient visit in the GPs’ private practice. GPs identify patients’ general eligibility and directly approach, inform, and pre-screen them based on specific inclusion and exclusion criteria (age, living situation, CFS, 10-m walking ability, medical conditions). If there is eligibility and willingness to participate after the pre-screening, written consent will be obtained by the GP to forward the pre-screening results and contact information to the respective study center, and to be contacted by the study staff for a subsequent telephone screening. In this context, GPs receive financial reward for recruitment, screening and referral of a potential participant. In the telephone screening, a study staff member obtains verbal informed consent and confirms pre-screening results of the GP and screens for the remaining exclusion criteria (800-m walking ability, German language skills, visual acuity). If there is still eligibility after the telephone screening, a first home visit is scheduled for the baseline assessment, and the written study information and consent form are mailed to the potential participant. At this visit, the assessor initially obtains written informed consent and screens for the final exclusion criterion of cognitive impairment, as defined by a Short Orientation-Memory-Concentration Test (SOMCT) score of > 10 [54, 83]. If there is no cognitive impairment (SOMCT score ≤ 10), all eligibility criteria are met and the home visit will be continued with the baseline assessment.

A secondary recruitment strategy will include advertising articles in local newspapers and site-specific AOK health magazines, and leaflets mailed to all AOK members aged ≥70 years at the study sites. Interested participants can contact the study centers, which will inform them about the study and refer them to their GP with an information sheet on the study procedures and eligibility criteria. GPs not yet participating can pre-screen patients for eligibility without study registration. The subsequent steps will be the same as for the primary recruitment strategy.

### Randomization and blinding

After the baseline assessment, participants will be randomly allocated to the IG or CG using computer-generated block randomization with a 1:1 allocation ratio stratified by study site and baseline CFS score. The randomization will be undertaken externally at the Institute for Epidemiology and Medical Biometry, Ulm University, Ulm, Germany using the randomization program ROM [84]. Participants will be informed about the randomization outcome by the physiotherapists at the phone call for scheduling the first home visit (IG: WEBB, CG: one-time counseling session). All primary and secondary outcome assessments will be undertaken by assessors blinded to group allocation. Data that identify group allocation such as training adherence (EARS, training diaries) will be collected by the unblinded physiotherapists.

### Data collection and management

A database management system will be used for data collection and management, in which all study staff involved in data collection will be trained in advance. Telephone screening data will be directly entered into the database. An electric case report form will be used for the baseline and follow-up assessments at participants’ homes, which is filled in offline and later uploaded into the database. Paper documents (e.g., training diaries, home visit documentation) will be used in machine-readable format and regularly transferred to the database manager. To ensure quality of data entry, minimize errors and missing data, the database management system includes automatic features for reminding data collection and detecting missing or implausible data entry. Individual identifiers and identifiable information of the participants will be kept on encrypted local servers at the three study sites as well as in the database, accessible only by authorized study staff and the external study monitor upon request. The pseudonymized final dataset will be accessible only to the study staff directly involved in the data analysis.

### Monitoring and harms

Study monitoring will be done by an external site monitoring institute (AMS – Advanced Medical Services GmbH, Mannheim, Germany) being entirely independent of the institutions and investigators involved in the study conduct. Systematic quality assurance and control will include a monitoring plan, regular on-site audits at every study center, partial source data verification, and site management. (Serious) adverse events/reactions will be monitored throughout the study to assess the safety of the trial and manage participant risk. Serious adverse events are defined as any harmful disease or injury that leads to hospital admission or death or resulted in persistent disability or incapacity. (Serious) adverse events (potentially) related to study participation will be reported to the ethic committees. ll participants enrolled in the study are covered by a subject and travel accident insurance.

### Statistical analysis

Group differences in the primary outcome of LLFDI-FC at month 12 (T_2_) will be analyzed in a confirmatory manner using a two-sided *t*-test for independent samples. In addition, linear regression analysis will be used to explore the group effect on LLFDI-FC at T_2_, adjusted for the baseline values and other covariates (e.g., age, sex, care need or comorbidities). Both analyses will also be conducted in an explorative manner after 6 months (T_1_) to evaluate time effects. Potential time effects over the total 12-month study period will be examined by analysis of covariance for repeated measures. The second, subordinated primary endpoint UAB-LSA will be analyzed using the same methods as described before for the LLFDI-FC. Secondary outcomes and safety issues at the T_1_ and T_2_ will be compared between the two treatment groups using *χ*^2^-tests or Fisher exact tests, Mann-Whitney *U* tests, *t*-tests for independent samples, multiple regression analyses as appropriate. To investigate whether specific subgroups (e.g., age, sex, functional baseline status) in IG benefit more from the PromeTheus program compared with their counterparts in CG, exploratory subgroup analyses will be performed by inclusion of interaction terms (subgroup × treatment) in each single statistical model. All main analyses will be conducted according to intention-to-treat principle, with all participants randomized after baseline assessment, regardless of subsequent treatment adherence, withdrawal from the study, or protocol deviation. Missing data will be imputed using multiple imputation by fully conditional specification method performed with SAS PROC MI. All statistical analyses will be performed with SAS version 9.4 (Statistical Analysis Software, SAS Institute Inc., Cary, North Carolina, USA) or R version 4.0.2 (R Foundation for Statistical Computing, Vienna, Austria).

## Discussion

The prevention and management of functional limitations and frailty in old age have become one of the most serious global public health challenges. Current evidence for effective intervention strategies to improve functional, mobility, and frailty status in community-dwelling (pre-)frail older adults is, however, still low [16, 23, 27, 85, 86]. It is becoming increasingly evident that multifactorial, interdisciplinary interventions are more effective than one-dimensional ones [23, 24, 87], and that physical activity plays a crucial role in this context [12, 22, 86]. There is an urgent need for large-scale and well-designed RCTs to evaluate the feasibility, implementation, and (cost-)effectiveness of community-based, multidimensional intervention strategies that make use of existing healthcare resources [16, 23, 34]. We expect to accrue more evidence-based knowledge about such an intervention strategy with this study.

As physical exercise has been identified as a key element for improving function and mobility in community-dwelling (pre-)frail older adults [12, 22, 86], the WEBB program will be implemented as an obligatory PromeTheus intervention component. In addition to the home visits suggested for implementation of the WEBB program [40], participants will receive phone calls from the physiotherapists to provide closer social support for exercise prescription and address barriers and motivational issues more frequently. Indeed, social reinforcement through phone calls has been shown to be an inexpensive and feasible method to increase adherence and effectiveness of home-based exercise programs in frail older adults [88].

Diverse facultative intervention components are initiated following standardized assessments of participants’ individual needs. As recommended [12, 16], to close the gap between research and clinical practice and to broaden the focus to the public health and system levels, the PromeTheus program integrates existing structures of the German healthcare system to implement these components.

An individual’s level of functioning (i.e., activity and social participation) can be influenced by contextual factors [89]. Removing environmental barriers through appropriate home modifications and provision of assistive devices can play an essential role in enabling older adults to “age in place”. Intervention components on person-environment-fit have thus been suggested to be included in comprehensive frailty interventions [16, 24, 90]. Home modifications and assistive devices have also been shown to be effective as single intervention components for maintaining and improving daily functioning, reducing the risk of falls, decreasing costs for personal assistance and healthcare, and increasing community participation in frail older adults [91–93]. Referral to local service providers for home modifications and to GPs for prescribing assistive devices will be initiated when there is a need and willingness of the participants.

Care needs of frail older adults are often complex and span across physical environmental and psychosocial domains. In community settings, insufficient psychosocial support has been identified as the most common self-perceived unmet care need of frail older adults [94]. Such support is therefore strongly recommended in clinical practice guidelines for frailty management [95]. As part of the interdisciplinary team of the PromeTheus intervention, social workers qualified also as long-term care counselors are integrated to offer to counsel on coping with everyday life with a special focus on psychosocial needs and social participation.

Insufficient nutritional intake and frailty are closely related [96]. Community-dwelling (pre-)frail older adults are often malnourished or at risk for malnutrition [97], and the co-occurrence of (pre-)frailty and poor nutrition substantially increases the risk for adverse health outcomes [98]. Participants identified as being malnourished or at risk for malnutrition are provided professional nutritional counseling. Physical exercise combined with nutritional interventions has shown to be particularly effective at reducing frailty and improving physical functioning in (pre-)frail older adults [24, 85–87].

Social isolation and loneliness have been linked to reduced physical functioning and frailty in older adults [17] and identified as risk factors for frailty onset and progression [19, 20]. To promote social participation and reduce loneliness, consideration will be given to referring participants to available local group activities as needed. In fact, social engagement in cultural group activities has been shown to decrease the risk for frailty [99].

We expect that the PromeTheus program will preserve physical function and mobility, reduce adverse behaviors (inactivity, malnutrition, social isolation), and improve contextual conditions, thereby leading to enhanced participation [89]. The LLFDI-FC as a PROM assesses self-reported physical functioning. Such person-centered outcomes providing information on what is meaningful from the perspective of the individual person have rarely been studied in frailty intervention trials, and their use has been recommended to address the current evidence-practice gaps for frailty management [16]. The LLFDI-FC has shown to have a higher predictive value for adverse health outcomes (e.g., hospitalization, falls, low self-reported health) than performance-based measures [71] and the Fried frailty phenotype [100] in older primary care patients. The LSA defined as a second primary outcome is a comprehensive measure of self-reported real-life mobility within the home and community. It assesses how far and often a person moves to different locations, also taking into account the use (or lack) of equipment or personal assistance. Considering the spatial context of mobility and the dependency on contextual factors, the LSA can also reflect participation in the society and the presence of adequate material and social environment [57, 101].

Robust evidence on the cost-effectiveness of frailty interventions is scarce [85], especially for multifactorial, interdisciplinary approaches in primary care based on existing healthcare structures [16]. An economic evaluation, as will be conducted in this study, has been recommended as one of the most urgently required strategies to close the gap between evidence and clinical practice [16]. Results of the cost-effectiveness analysis will indicate whether the effacts gained by the PromeTheus program outweigh the associated costs.

The study also includes a process evaluation to collect information that will be essential for further large-scale implementation of the PromeTheus program into routine care. Results of the process evaluation will provide a detailed understanding of facilitating and hindering factors for the delivery and outcomes of the complex program, enhance the external validity of the study findings, and identify recommendations for potential adaptions to increase the likelihood of successful implementation into routine care. All this will contribute to increasing the value of the evidence generated by the study for policy and practice [15].

In summary, this study will provide insight into the (cost-)effectiveness and implementation of a multifactorial, interdisciplinary intervention designed to prevent functional and mobility decline in (pre-)frail older adults in the community. If the PromeTheus program is shown to be (cost-)effective, there are major potential benefits to (pre-)frail older adults and the healthcare system. Preventing physical and mobility decline could reduce adverse health outcomes, such as frailty, disability, social isolation, and poor quality of life [1, 17, 102–105], and associated healthcare costs [8, 9, 106]. We thus expect that preserving physical functioning and mobility will contribute to maintain independence, reduce the needs and costs for care, and enable more participation and greater quality of life. As the PromeTheus program makes use of healthcare structures already available in all federal states (e.g., GPs, physiotherapists, service providers with counseling services), it is readily transferable to German healthcare for (pre-)frail older adults on a large scale.

### Trial status

Registration of GPs to participate in the PromeTheus study has been ongoing since January 2021.The official start of participant recruitment was May 2021; enrollment of the last participant is planned for April 30, 2022. By the time of submission (November 2, 2021), *n* = 86 participants have already been enrolled in the trial.

## Data Availability

The de-identified datasets generated and/or analyzed during the current study may be made available from the corresponding author upon reasonable request once the study has been completed.

## Abbreviations

AOK: ‘Allgemeine Ortskrankenkasse’ (health insurance company)
CFS: Clinical Frailty Scale
CG: control group
DEMMI: de Morton Mobility Index
EARS: Exercise Adherence Rating Scale
FIMA: ‘Fragebogen zur Inanspruchnahme medizinischer und nicht-medizinischer Versorgungsleistungen im Alter’ (questionnaire on the use of medical and non-medical care services in old age)
FIT: Frailty Intervention Trial
GP: general practitioner
IG: intervention group
LLFDI: Late-Life Function and Disability Instrument
LLFDI-FC: Late-Life Function and Disability Instrument – Function Component
LSA: Life-Space Assessment
MNA-SF: Mini Nutritional Assessment – Short Form
PROM: person-reported outcome measure
SNAQ: Simplified Nutritional Appetite Questionnaire
SOMCT: Short Orientation-Memory-Concentration Test
TIDieR: Template for Intervention Description and Replication
WEBB: Weight-bearing Exercise for Better Balance
